# Advanced prodrug strategies in nucleoside analogues targeting the treatment of gastrointestinal malignancies

**DOI:** 10.3389/fcell.2023.1173432

**Published:** 2023-04-18

**Authors:** Xingxing Xu, Zixuan Li, Xueying Yao, Nannan Sun, Junbiao Chang

**Affiliations:** School of Pharmaceutical Sciences, Zhengzhou University, Zhengzhou, China

**Keywords:** gastrointestinal malignancies, anticancer, nucleoside analogue, prodrug strategy, ProTide

## Abstract

Gastrointestinal malignancies are common digestive system tumor worldwide. Nucleoside analogues have been widely used as anticancer drugs for the treatment of a variety of conditions, including gastrointestinal malignancies. However, low permeability, enzymatic deamination, inefficiently phosphorylation, the emergence of chemoresistance and some other issues have limited its efficacy. The prodrug strategies have been widely applied in drug design to improve pharmacokinetic properties and address safety and drug-resistance issues. This review will provide an overview of the recent developments of prodrug strategies in nucleoside analogues for the treatment of gastrointestinal malignancies.

## 1 Introduction

Gastrointestinal (GI) cancers include colorectal, gastric, oesophageal, pancreatic, and liver cancer, which continue to be a significant and common cause of mortality and morbidity worldwide. Among them, colorectal and gastric cancers rank second and fourth in global mortality respectively (Sung et al., 2021). In the United States, about 153,020 individuals will be diagnosed with colorectal cancer (Siegel et al., 2023) and 26,500 new cases of stomach cancer (American Cancer Society, 2023) in 2023. It is no doubt that radiotherapy, surgery, chemotherapy, targeted therapy, and immunotherapy all have been proven effectiveness in the treatment of GI malignancies, there still remains unmet need for new drugs and therapies to protect the patient from relapse and ensure long-term survival.

**FIGURE 1:**
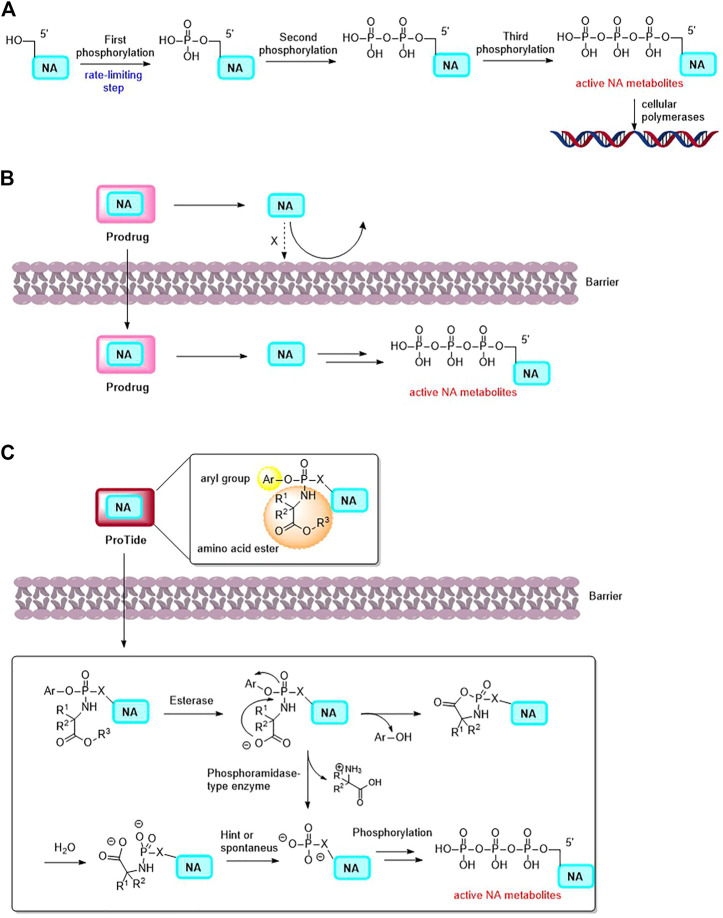
**(A)** The activation mechanism of NA (NA: nucleoside analogue). **(B)** The concept of Prodrug. Prodrug is able to deliver the parent drug (NA) to specific tissue. **(C)** Postulated mechanism of the mechanism of ProTides to release NA and its active metabolites.

Nucleoside analogues (NAs, composed of nucleobases and ribose or deoxyribose) and nucleobases are invaluable components for various malignancies chemotherapy treatment. To induce cytotoxicity, these compounds act as antimetabolites, competing with physiological nucleosides, and interacting with a multitude of intracellular targets (Galmarini et al., 2001; Galmarini et al., 2002; Acharjee, 2020). After a series of phosphorylation, nucleoside-5′-triphosphate analogs are generated, and they can be incorporated into DNA as DNA polymerases substrates during DNA replication or excision repair synthesis. As a result, cell progression is terminated and that leads to apoptosis (Figure 1A) (Jackson and Bartek, 2009; Shelton et al., 2016). Due to the fact that cancer cells duplicate their genomes often more frequently than normal cells, it is possible for the enrichment of nucleoside-5′-triphosphate analogs in cancer cells (Parker, 2009; Counihan et al., 2018). The use of nucleoside analogue drugs, however, has some limitations: 1) NAs are structurally different from natural nucleosides, therefore, they are often inefficiently phosphorylated, which limits the active triphosphate metabolite production (Thornton et al., 2016; Fàbrega et al., 2021; Zhang et al., 2022). 2) As polar molecules, NAs and nucleobases also have poor oral bioavailability due to their low permeability (Li et al., 2008; Yan et al., 2023). 3) A number of complex mechanisms are involved in the development of NAs to avoid drug resistance and toxicity, such as the dephosphorylation of active NA metabolites by phosphatases, or the inactive metabolite of NAs by increased catabolic bioconversion. Facing the challenges of anticancer NAs research, a good way is to developing prodrugs of NAs which could circumvent some of the parent NA drug disadvantages.

It was successfully demonstrated that nucleotide prodrugs could overcome above issues. NA prodrugs decrease drug resistance by avoiding certain routes of catabolic bioconversion and increase nucleotides oral absorption (Thornton et al., 2016; Acharjee, 2020; Yan et al., 2023). Moreover, nucleotide prodrugs can deliver nucleotides to a specific tissue *in vivo*, changing their selectivity and reducing their toxicity (Figure 1B) (Li et al., 2008; Seley-Radtke and Deval, 2018). Phosphonate-containing NA prodrugs have also been investigated, as well as isosteric and isoelectronic phosphonates, to cross the monophosphorylation barrier and improve half-lives of the nucleoside monophosphonates as compared to their monophosphates (Thornton et al., 2016). Among these prodrug approaches, the ProTide (Pro-Nucleotide, the NA monophosphate or monophosphonate groups are masked by aromatic groups and amino acid esters, which could be cleaved off inside cells to release free nucleoside monophosphate and monophosphonate. Figure 1C) prodrug technology is notable, that was pioneered by Prof. Chris McGuigan (Cardiff, United Kingdom) (Mehellou et al., 2009; Mehellou, 2016; Mehellou et al., 2018). A number of anticancer NA prodrugs have been developed using ProTide technology. In this review, focusing on the chemical structures and the application of prodrug strategies, we discuss recent developments in NA prodrugs for GI malignancies treatment.

## 2 Nucleoside analogues for the treatment of GI malignancies

Chemotherapy is still the main treatment for GI malignancies. In current guidelines, perioperative chemotherapy or postoperative chemotherapy plus chemoradiation are listed as preferred approaches, although chemotherapy after surgery is also an option (Joshi and Badgwell, 2021). Nucleoside analogues, as 5-fluorouracil (5-FU) and gemcitabine, represent an integral component of recommended first-line GI malignancies treatment regimens. Some novel NAs also demonstrate potentials in GI cancer. Azvudine is one of these NAs, a notable cytidine analogue having both anti-HIV and anti-SARS-CoV-2 activities, but also having anticancer activities. The azvudine treated gastric cancer xenografts mouse (SGC7901) demonstrated considerable growth inhibition activity in a dose-dependent manner with lower toxicity (the antitumor effect of 2.0 mg/kg azvudine is comparable with 400 mg/kg capecitabine) (Wang et al., 2011; Fayzullina et al., 2022). Though NAs have showed their anticancer potency, the emerging issues, like drug resistance and low drug concentration at the tumor site, block the NAs application of anti-GI cancer. Prodrug strategies of NAs, particularly the ProTide approach, could address the emerging problems with promising future in oncology. The prodrug of 5-FU and gemcitabine are the most advanced NA prodrugs for the treatment of GI malignancies.

### 2.1 Prodrugs of 5-FU

5-FU is an essential nucleobase which has been frequently administered to GI cancer patients since 1957 (Verma et al., 2022) (Table 1). Bearing a fluorine atom at position 5 of uracil, 5-FU is catalyzed by the corresponding enzyme as active metabolites that could incorporate into RNA (5-FU-5′-triphosphate, FUTP) and DNA (2′-deoxyribose-5-FU-5′-triphosphate, FdUTP) and inhibit the nucleotide synthetic enzyme thymidylate synthase (Longley et al., 2003; Sethy and Kundu, 2021). Although the efficacy of 5-FU makes it one of the most widely used chemotherapy agents, the phosphorylation of 5-FU in the digestive tract causing myelosuppression and gastrointestinal disorders, restricted cellular uptake and complex enzymatic hydrolysis processes directly contribute to drug resistance, the poor oral bioavailability limits the administration mode of 5-FU, it has a short half-life (*t*_1/2_: 8–20 min) and is rapidly eliminated from the plasma (Sethy and Kundu, 2021). Besides, intravenous injection brings the risk of venous thrombosis or infection round the catheter (Grem, 2000; More et al., 2021). Thus, developing prodrugs of 5-FU that diminish or circumvent some of these disadvantages is a major challenge.

**TABLE 1:** The structures of NAs for the treatment of GI malignancies.

Name	Other names	Structure	Indications in GI malignancies/Dosage form
5-Fluorouracil	5-FU	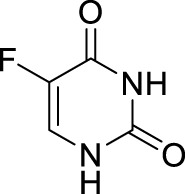	Colorectal, oesophageal, stomach, pancreatic cancers/Intravenous injection
Doxifluridine	5′-dFUrd; 5′-DFUR; 5′-deoxy-5-fluorouridine; Doxyfluridine; Furtulon; Ro 21-9738	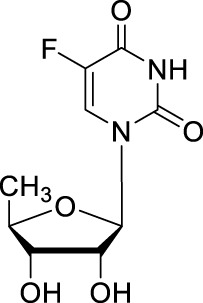	Stomach, colorectal, bile duct, pancreatic cancers, hepatocarcinoma/Oral
Capecitabine	CAP, Ro 09-1978	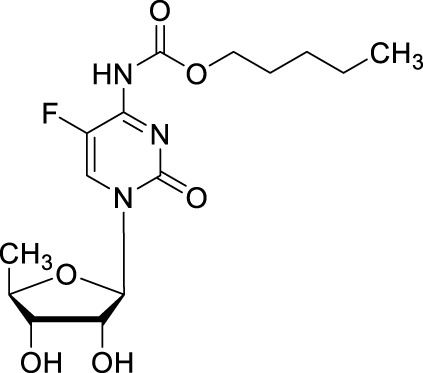	Colorectal, pancreatic, stomach, esophageal, gastroesophageal junction cancers/Oral
NUC-3373	NUC-3073, CPF-373	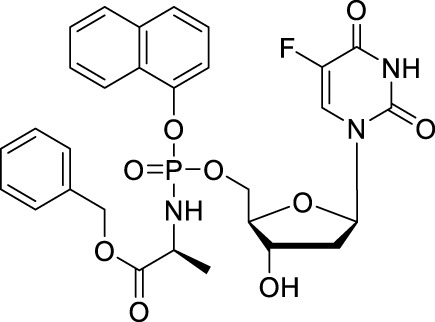	Colorectal, pancreatic cancers/Intravenous infusion
Gemcitabine	2′,2′-difluoro-2′deoxycytidine, dFdC, GCB	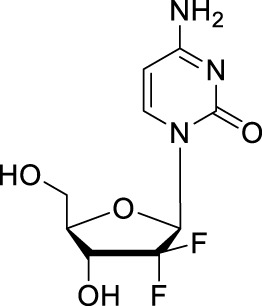	Bile duct, pancreatic cancers, hepatocarcinoma/Intravenous infusion
LY2334737	LY-2334737	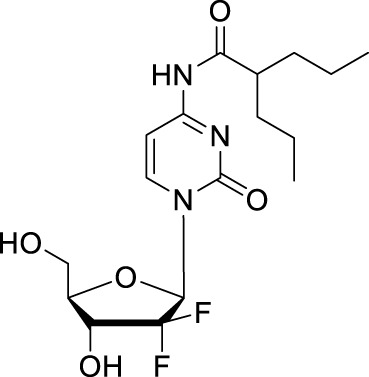	Colorectal, pancreatic cancers/Oral
CP-4126	CO-1.01, CO-101	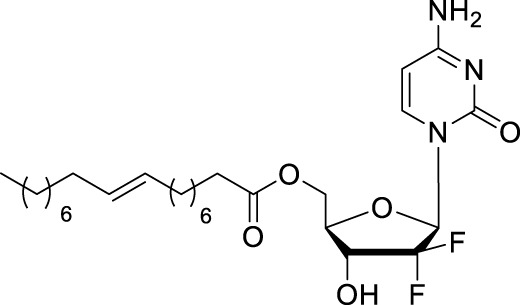	Colorectal, pancreatic cancers/Oral
NUC-1031	Acelarin, CPF-31, MTL-007, GTPL7389	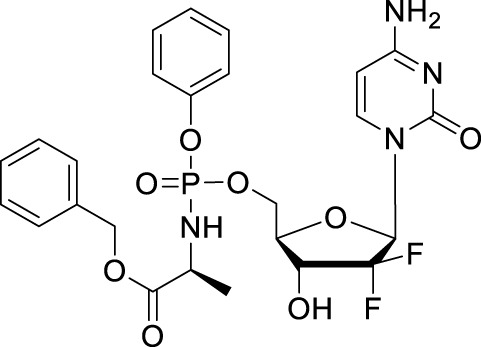	Colorectal, bile duct, pancreatic cancers/Oral

#### 2.1.1 Doxifluridine

5′-Deoxy-5-fluorouridine (5′dFUrd, doxifluridine, Table 1), an orally available prodrug of 5-FU, was synthesized by Cook *et al.* in 1979 (Cook et al., 1979). To avoid degradation in gut and phosphorylation in digestive tract, researchers at Roche used prodrug strategies by adding a pseudo pentose is bonded in position 1 of 5-FU to develop an orally available prodrug doxifluridine (Zampino et al., 1999). As missing the 5′-hydroxyl groups, doxifluridine cannot be phosphorylated and thus cannot be directly metabolized in DNA or RNA synthesis. *In vivo*, doxifluridine is converted to 5-FU by either thymidine phosphorylase or pyrimidine-nucleoside phosphorylase (Kono et al., 1983; Nishioka et al., 2007). Pyrimidine nucleoside phosphorylase is highly expressed in patients with colorectal cancer and esophageal squamous cell carcinoma, and especially in tumor tissues such as esophageal, pancreatic, and hepatic, indicating a higher tumor specificity and better safety of doxifluridine than that of 5-FU (Taguchi, 1987; Noguchi et al., 2003; Ogata et al., 2007).

At the beginning of developing doxifluridine, intravenous administration of doxifluridine can produce comparable tumor-free survival while the systemic exposure to the cytotoxic metabolite 5-fluorouracil is minimized. Then the first clinical trials of doxifluridine was by *i.v.* administration and has indicated good activity for colorectal cancers, however, toxic effects on the nervous system and the cardiovascular system prevents this mode of administration (Van Der Heyden et al., 1999). The oral bioavailability of doxifluridine was 34%–47%, which was more satisfactory than that of 5-FU, and with a comparably show elimination half-life (*t*_1/2_ of 32–45 min) (Van Der Heyden et al., 1999; Vivian and Polli, 2014). Besides, oral administration of doxifluridine can avoid the neurological and cardiac complications caused by *i.v.* administration. Because gastrointestinal tracts rich in thymidine phosphorylase, the dose-limiting toxicity of orally dosed doxifluridine was found in human intestinal tract (nausea, diarrhea) (Lamont and Schilsky, 1999). Doxifluridine is used as a cytostatic agent in chemotherapy in several Asian countries including Japan, China and South Korea. In clinical trials, doxifluridine has also been administered intravenously either as a monotherapy or in combination with mitomycin-C and cisplatin.

#### 2.1.2 Capecitabine

To circumvent the unacceptable gastrointestinal toxicity of doxifluridine, researchers at Roche continually developed a series of *N* ^4^-substituted 5′-deoxy-5-fluorocytidine derivatives, which could be hydrolyzed to doxifluridine and 5-FU. As a result, *N* ^4^-pentyloxycarbonyl-5′-deoxy-5-fluorocytidine (capecitabine, CAP, Table 1) stood out by such prodrug strategy as an orally chemotherapeutic drug that was FDA-approved for the treatment of colorectal cancer, pancreatic adenocarcinoma, stomach cancer, esophageal cancer, or gastroesophageal junction cancer (Siddiqui et al., 2019; Pouya et al., 2021; Ge et al., 2023). As mentioned above, the gastrointestinal toxicity of doxifluridine is attributed to the liberation of 5-FU in intestine tract under the action of thymidine phosphorylase, capecitabine was thus designed as a prodrug of doxifluridine that could not be the substrate for thymidine phosphorylase in the intestine with an oral bioavailability of about 100%, C_max_ of 3.9 mg/L, *t*_max_ of 1.5–2 h, and AUC of 5.96 mg·h/L (Walko and Lindley, 2005; Rehman et al., 2022). Capecitabine is rapidly and intently absorbed after oral administration, then it is cleaved by liver carboxylesterases after passing through the intestinal mucosa and catalyzed by cytidine deaminase to form doxifluridine in either liver or tumor, then capecitabine is subsequently converted to 5-FU by thymidine phosphorylase (Ishitsuka, 2003). Diarrhea, the typical toxicity issue caused by doxifluridine, could be well avoid by capecitabine because of the intact across of the gastrointestinal barrier.

Comparing to doxifluridine or 5-FU, capecitabine was highly effective in CXF280, HCT116 and COLO205 human colon cancer xenograft models with higher levels of parent drug 5-FU in tumor than in plasma (114- to 209-fold) and muscle (21.6-fold) (Ishikawa et al., 1998). In colorectal cancer patients, after capecitabine was orally administered with 1,255 mg/m^2^ of twice daily over 5–7 days, the concentration of parent drug 5-FU was on average 3.2 times higher in primary colorectal tumors than in adjacent healthy tissue, 21 times greater than in plasma (Li et al., 2023). Clinical studies showed that a statistically significant improvement in the overall response rates was observed and the safety profile was in favor of capecitabine (Beenet, 2021; Bryson et al., 2021). Capecitabine has been approved by FDA to be used alone or with other drugs to treat GI cancers and is now commercialized in Europe as a monotherapy for first-line treatment of metastatic colorectal cancer. In brief, using prodrug strategy, capecitabine was developed with increased concentration at the tumor site and decreased concentration in healthy tissues with a consequent reduction in systemic toxicity.

#### 2.1.3 NUC-3373

Though the prodrugs of 5-FU have achieved clinical success as anticancer agents, acquired drug resistance have been observed because of reduced levels of the activating enzyme (phosphorylating FdUTP), overexpression of thymidylate synthase (the main target for 5-FU and FUDR), upregulation of the degradative enzyme thymidine phosphorylase (TP) and reduced transporter-mediated entry of nucleoside/nucleobase (5-FU or FdUTP) into cells (Parker, 2009; Kumar et al., 2022). Nucleotides could overcome these matters, but since nucleotides are poorly membrane soluble and readily dephosphorylated, a better approach for solving this drug resistance is to dissimulate the monophosphate as a phosphate prodrug. In 2011, McGuigan *et al.* reported phosphate prodrugs of 5-FU and FUDR by phosphorochloridate chemistry with variation in the aryl, ester, and amino acid regions (McGuigan et al., 2011). NUC-3373 is one of the phosphoramidate prodrugs (Table 1).

The cytotoxic activity of NUC-3373 can be achieved without thymidine kinase in TK-deficient tumor cells, and NUC-3373 remains resistant to degradation by catabolic enzymes including TP and dihydropyrimidine dehydrogenase (DPD) (Vande Voorde et al., 2011). NUC-3373 produced more FdUMP (active metabolite of 5-FU, 5-fluoro-2-deoxyuridine monophosphate) than 5-FU (366-fold) in human colorectal cancer cell line HT29 and NUC-3373 showed greater tumor reduced volume than 5-FU in HT29 xenograft studies (Ghazaly et al., 2017). PK studies demonstrated that NUC-3373 has a long plasma half-life (*t*_1/2_ = 9.7 h), which is significantly improved compared to 5-FU. A Phase II study of NUC-3373 in combination with standard agents used in colorectal cancer treatment (ClinicalTrials.gov Identifier: NCT05678257) is ongoing and a Phase I clinical trial for treatment of advanced solid tumors has been complete.

### 2.2 Prodrugs of gemcitabine

Gemcitabine is a pyrimidine nucleoside analog (2′,2′-difluoro-2′deoxycytidine, Table 1), which isoriginally synthesized by Hertel and colleagues at Eli Lilly (Hertel et al., 1990). Gemcitabine is another useful chemotherapy for GI cancer, especially is used as a first-line treatment alone for pancreatic cancer. Gemcitabine in combination with cisplatin is currently being investigated as an adjuvant therapy for biliary tract cancers (Oh et al., 2022; Beutel and Halbrook, 2023; Ioka et al., 2023). But there are still challenges associated with gemcitabine’s long-term use in cancer therapy. The main challenges include the inactive deaminated metabolite 2′,2′-difluoro-2′-deoxyuridine (dFdU), which is deaminated by cytidine deaminase at the *N* ^4^ position of the cytidine base in the serum, and dFdU-5′-monophoshate, which is deaminated by deoxycytdine deaminase in the cell (Serdjebi et al., 2015; Shelton et al., 2016; Miao et al., 2020). Prodrug strategies have also been applied for developing novel NAs based on gemcitabine.

#### 2.2.1 LY2334737

LY2334737 was designed by blocking the deamination site at the *N* ^4^ position of gemcitabine (Table 1). In 2009, Bender and collaborators introduced a valproic acid to the *N* ^4^ position of the cytosine to improve the orally bioavailability of gemcitabine (Bender et al., 2009; Zhou et al., 2018). The enzymatic stability of LY2334737 is remarkable. A study showed that LY2334737 is insensitive about many hydrolases, only carboxylesterase-2 hydrolyzed LY2334737 to gemcitabine and required long incubation periods of 0.5–2 h (Pratt et al., 2013). Prodrugs with an amide linkage to valproic acid, instead of ester linkages, are likely to exhibit slow cleavage rates. With oral dosing, this prodrug passes through the intestinal epithelium and is absorbed intact and gemcitabine is formed slowly in the body. As in a mice PK study, orally administered LY2334737, the systemic exposure (AUC) of gemcitabine was 778 ng·h/mL with *t*_max_ of 1 h, which was higher and longer compared with the value that was under the same molar dose of oral gemcitabine (AUC of 536 ng·h/mL, *t*_max_ = 0.5 h) (Bender et al., 2009). Additionally, mice treated with LY2334737 had a less than half ratio of dFdU to gemcitabine than mice given an equal dose of gemcitabine, demonstrating the possibility of *N* ^4^-modification strategy for new nucleoside-based prodrug discovery.

#### 2.2.2 CP-4126

CP-4126 (CO-1.01, Table 1) is a 5′-elaidic acid ester prodrug of gemcitabine developed by Clavis Pharma. The fatty acid ester chain could increase the ability to diffuse through the plasma membrane and avoid the resulting drug resistance. As a result of this modification, CP-4126 is a transporter-independent analog of gemcitabine, entering cells primarily without hENT1 (Galmarini et al., 2009; Alexander et al., 2016). Besides, the 5′-elaidic acid ester of CP-4126 could effectively protected gemcitabine from deamination (Bergman et al., 2011). Thus, it was suggested that CP-4126 had efficacy as a second-line treatment in gemcitabine-resistant pancreatic adenocarcinoma patients with negative tumor hENT1 expression. Safety and efficacy of CP-4126 have been evaluated in several Phase I/II clinical trials, including metastatic pancreatic adenocarcinoma (ClinicalTrials.gov Identifier: NCT01124786), gemcitabine-resistant pancreatic adenocarcinoma (ClinicalTrials.gov Identifier: NCT01233375) and other advance solid tumors (Stuurman et al., 2013). Whereas, the primary endpoint of this study was not met, and no efficacy signal was identified for CP-4126 in treating pancreatic adenocarcinoma with progressive metastatic disease (Li et al., 2014).

#### 2.2.3 NUC-1031

Though 5′-ester prodrugs CP-4126 improved metabolic properties, it is still failed to show superiority over gemcitabine during clinical trials. In 2014, McGuigan’s research team used the ProTide technology to design a series of 5′-phosphoramidate gemcitabine prodrugs in order to overcome the mechanisms of cancer cell resistance that limit the clinical treatment of gemcitabine, within these agents, NUC-1031 is the most potent one (Table 1) (Slusarczyk et al., 2014). NUC-1031 could avert gemcitabine resistance mainly benefit from its unique ProTide characteristic. First, NUC-1031 effectively avoid the deamination of cytidine deaminase because of the presence of the phosphoramidate moiety on the 5′-O-position (Kapacee et al., 2020). Besides, this ProTide was lipophilic enough for passive diffusion into cells and the cellular uptake of NUC-1031 was not dependent on nucleoside transporters (McNamara et al., 2020). In addition, the 5′-phosphoramidate structure of the ProTide is able to bypass the rate-limiting step of the monophosphorylation by deoxycytidine kinase which is downregulated in gemcitabine-resistant cancer cells (Boyd et al., 2020). The pharmacokinetic analysis of NUC-1031 revealed that it had a significantly longer half-life than gemcitabine, with a plasma *t*_1/2_ of 9.7 h (Kapacee et al., 2020). It was also observed that NUC-1031 was rapidly absorbed intracellularly by peripheral blood mononuclear cells. NUC-1031 (*C*_max_ was 764 pmol/10^6^ cells/h with a *t*_max_ of 20 min) will be successfully converted to the active moiety dFdCTP with a *C*_max_ of 727.5 pmol/10^6^ cells at the 500 mg/m^2^ equivalent dose after 30 min post end of infusion. NUC-1031 also demonstrated potent intracellular concentrations of the active gemcitabine triphosphate metabolite that was 13 times higher than achieved by gemcitabine (Mohanty et al., 2018). In terms of safety and tolerability, NUC-1031 was comparable to gemcitabine, with similar side effects and serious adverse events (SAEs) (Kapacee et al., 2020).

NUC-1031 was studied *in vivo*, particularly in gemcitabine resistant pancreatic cancers. After administration of NUC-1031 by 7 days, the tumor size reduced faster and more significantly than that in dosed gemcitabine, and NUC-1031 showed good tolerance with less than 4% reduction in body weight over the treatment (Slusarczyk et al., 2014). The clinical study of NUC-1031 in metastatic pancreatic carcinoma (a Phase III, open label, multicentre randomised clinical study comparing NUC-1031 with gemcitabine in patients with metastatic pancreatic carcinoma, ClinicalTrials.gov Identifier: NCT03610100) is attractive, but now, it is suspended to recruitment following a review on efficacy and toxicities.

## 3 Conclusion

Developing NAs is important for the discovery of chemotherapy agents. Prodrug strategies to discover new NAs are widely used, particularly the ProTide strategy. With the ProTide technology, novel nucleoside monophosphate analogues have been discovered and applied to the clinic in recent years, and there have been two FDA-approved ProTides (tenofovir alafenamide and sofosbuvir) as well-known antiviral drugs. This prodrug technology has already been proven effective in delivering the activated parental drug into cells avoiding drug resistance and toxicity. However, it is important to note that nucleoside monophosphate analogues with their stereocenter at the phosphorus atom may cause different biological activities and potencies of enantiomers or diastereomers (Slusarczyk et al., 2021). Developing diastereoselective synthetic and separation methods for ProTide is therefore necessary. In summary, in light of the applications of prodrug technology, it is possible that new NA ProTides will be developed for treating cancers, especially GI malignancies, with improved efficacy and avoidable drug resistance.
